# EFFECTS OF DEMENTIA DIAGNOSIS ON HOSPITAL READMISSION AFTER DISCHARGE FROM A SKILLED NURSING HOME

**DOI:** 10.1093/geroni/igae098.3075

**Published:** 2024-12-31

**Authors:** Jennifer Carnahan, James Slaven, Emily Fox Ludden, Corinne Bowditch, Healey Ryan, Li Jiangqiong, Wanzhu Tu, Alexia Torke

**Affiliations:** Indiana University/Regenstrief Institute, Indianapolis, Indiana, United States; Indiana University Indianapolis, Indianapolis, Indiana, United States; Regenstrief Institute, Inc., Indianapolis, Indiana, United States; Oakland University William Beaumont School of Medicine, Rochester, Michigan, United States; Indiana University Indianapolis, Indianapolis, Indiana, United States; Indiana University Indianapolis, Indianapolis, Indiana, United States; Indiana University Indianapolis, Indianapolis, Indiana, United States; Indiana University School of Medicine, Indianapolis, Indiana, United States

## Abstract

High acute care utilization has been associated with dementia, but the risk of repeat acute care use following a skilled nursing home (SNH) to home transition is not well understood. We examined the association of dementia with 30-day hospital readmission after leaving a SNH. Data from the Health and Retirement Study from 2000-2018 was linked to Medicare and SNH claims. We analyzed the events of hospital readmission following SNH discharge using logistic regression while controlling for repeated within-subject readmissions. There were 5,912 discharges to the community from an SNH and 941 hospital readmissions. ICD codes for dementia were present with 1,754 of the SNH-to-home transitions and 314 of the hospital readmissions. In bivariate analysis, people with dementia were more likely to be readmitted (p=0.0074). Post-SNH readmissions were also more likely among people who were Black, male, dual eligible beneficiaries, longer pre-SNH hospital stays, higher Charlson Comorbidity Index, and greater activities of daily living deficits. In the multivariable logistic regression model, the odds of readmissions were still greater among persons with dementia, but significance was attenuated (OR 1.09; 0.90, 1.32; p=0.4065). Significant associations with readmissions include the Charlson Comorbidity Index (OR 1.07; 1.04, 1.09; p< 0.0001) and activities of daily living deficits (OR 1.11 (1.04, 1.17; p=0.0005). People with dementia may be at greater risk of readmission after SNH discharge but not when controlling for factors such as socioeconomic status, activities of daily living, and other comorbidities.

